# From NELA to EPOCH and beyond: enhancing the evidence base for emergency laparotomy

**DOI:** 10.1186/s13741-016-0048-x

**Published:** 2016-09-01

**Authors:** Peter M. Odor, Michael P. W. Grocott

**Affiliations:** 1 University College London Hospital, 235 Euston Rd, London, NW1 2BU UK; 2 Anaesthesia and Critical Care Research Unit, University Hospitals Southampton NHS Foundation Trust, CE.93 Mailpoint 24, E-Level Centre Block, Southampton, SO16 6YD UK; 3 Critical Care Research Area, NIHR Respiratory Biomedical Research Unit, University Hospital Southampton NHS Foundation Trust, Southampton, SO16 6YD UK; 4 Integrative Physiology and Critical Illness Group, Clinical and Experimental Sciences, Faculty of Medicine, University of Southampton, University Hospital Southampton NHS Foundation Trust, Tremona Road, Southampton, SO16 6YD UK; 5 National Institute of Academic Anaesthesia Health Services Research Centre, Royal College of Anaesthetists, London, WC1R 4SG UK

**Keywords:** National Emergency Laparotomy Audit, Evidence-based medicine, Emergency surgery

## Abstract

Around 35,000 patients undergo emergency laparotomy surgery in the UK each year with an in-hospital 30-day mortality estimated as between 11 and 15 %. The recent publication of the First Patient Report of the National Emergency Laparotomy Audit (NELA) has provided a detailed description of individual hospital performance against national standards of care in emergency laparotomy in England and Wales. Although the standards used for audit purposes in NELA are based upon the best currently available evidence, none of the source data derives from randomised controlled studies. This commentary explores the evidence base for the standards evaluated by NELA and highlights recent and forthcoming studies that may substantially contribute to improving the evidence base in this area, thereby improving patient care and strengthening the validity of the NELA audit standards.

## Background

The First Patient Report of the National Emergency Laparotomy Audit (NELA) was launched on 30 June 2015 (NELA project team 2015). Data from over 20,000 patients, across 192 National Health Service hospitals in England and Wales, informed the most comprehensive review of perioperative care for emergency surgery to date. This commentary highlights the limitations of the evidence underpinning the standards against which NELA evaluates the care of patients undergoing emergency laparotomy (EL) and highlights some important recent and forthcoming studies that will both improve patient care and feed into the process of updating the standards against which NELA audits process and outcome.

## Main text

Central to understanding of the First Patient Report of NELA (NELA project team 2015) is an appreciation of the methodological framework around which the project is based. NELA was not designed to address a specific research hypothesis but rather to provide a powerful resource of structure, process and outcome measures to drive quality improvement in the care of patients undergoing EL in the England and Wales. Key standards of care for EL patients were identified from national guidelines and policy documents and were then used to define questions answerable by audit of individual hospital performance. The national picture provided by NELA on the size and nature of problems surrounding perioperative care of EL and the provision of high-quality risk-adjusted outcomes is in itself valuable. More importantly, NELA provides benchmarking process and outcome data to drive local quality improvement initiatives that should improve patient outcomes. Yet, much like the Hip Fracture Anaesthesia Sprint Audit of Practice (ASAP) (ASAP collaboration team 2014), the design of NELA, imposed by the audit framework, is also a limitation: the constraint of only being able to collect data linked to existing standards of care.

It is therefore relevant to consider the quality and grade of evidence underpinning the auditable standards applied within NELA. Nine of the 19 chapters in the First Patient Report contain recommendations, linked to a total of 22 standards, which are in turn derived from 9 different standards documents. The standards documents fall into a relatively limited number of categories; three are reports of the National Confidential Enquiry into Patient Outcome and Death (NCEPOD) (NCEPOD 2007; NCEPOD 2010; NCEPOD 2011), two are consensus statements from the Association of Surgeons of Great Britain and Ireland (ASGBI) (ASGBI 2007; ASGBI 2009), two are outputs from the Royal College of Surgeons of England (RCS) (RCSEng and Department of Health 2011; RCSEng 2011) (one guidance document and one report from a collaborative working group with the Department of Health (DoH)) and two are DoH or National Health Service England (NHSE) documents (Department of Health 2001; CQUIN 2015). However, despite the importance and influence of these national documents that are the sources of the standards for NELA, not one of the recommendations used as an auditable standard is underpinned by evidence from a study with a randomised controlled trial (RCT) design.

For example, chapter seven in the First Patient Report of NELA contains the auditable standard that “Patients admitted as an emergency should be seen by a consultant at the earliest opportunity. Ideally this should be within 12 hours and should not be longer than 24 hours”. This standard is taken directly from the NCEPOD Emergency Admissions report from 2007 (Emergency Admissions: A journey in the right direction 2007). NCEPOD reports are highly regarding for shaping clinical care in the UK. However, from a critical perspective, the NCEPOD methodology of post hoc review by a panel of experts of a non-control matched series of cases constitutes a low grading of evidence, with a lack of blinding and inherent risk of reporting and observer bias. The standard that “All high risk patients should be considered for critical care and as minimum, patients with an estimated risk of death of ≥10 % should be admitted to a critical care location.” which is taken from “The Higher Risk General Surgical Patient” report published jointly by the RCS and DoH in 2011 is unreferenced: trials of the efficacy or effectiveness of critical care and notoriously challenging and randomization may not be feasible. Whilst these standards make intuitive sense to the practitioner and are likely to improve patient outcome, we cannot know this for sure. Moreover, such changes may have significant resource implications (e.g. requirement for additional critical care beds) and carry the risk of unintended consequences. For example, admission of lower risk patients to Intensive Care following EL may divert resources from other patients that might have been admitted to the same bed and may have gained more benefit from that admission than the EL patient. Such dilemmas are the bread and butter of clinical decision-making, but when this decision-making is constrained by external influence of national standards, the importance of ensuring that these standards are based on the highest quality of available evidence is clear.

Similar limitations pertain for all the other NELA standards. Even the most strongly evidenced standard—that relating to antibiotic prescription in septic patients—is not based upon RCT evidence. The standard covers “the number of patients who present to emergency departments and other wards/units that directly admit emergencies with severe sepsis, Red Flag Sepsis or Septic Shock who received intravenous antibiotics within one hour of presenting.” This standard is derived from the NHSE 2015–16 Commissioning for Quality and Innovation (CQUIN) guidance (CQUIN 2015) and is supported by several references. Three of these references are observational studies describing sepsis prevalence but are unrelated to the timing of antibiotics (Vincent et al. 2006; Hall et al. 2011; Intensive care national audit and research centre 2015); three are parliamentary reports or NHSE Patient Safety Alerts on the magnitude of sepsis as a problem (Parliamentary and Health Service Ombudsman 2013; Parliamentary and Health Service Ombudsman 2014; NHS England 2014); and the final report comments on the cost and impact of sepsis (Esteban et al. 2007). The Royal College of Physicians Acute Care Toolkit on Sepsis (Acute Care Toolkit 9: Sepsis 2014) is also referenced and contain further sources, but again, only one (Kumar et al. 2006) of the four observational studies cited (Vincent et al. 2006; Trzeciak et al. 2006; Daniels et al. 2011) contains any detail on timing of antibiotics. Whilst this single retrospective study presents a convincing pattern of results and a strong treatment effect, which is compatible with a credible pathophysiological mechanism, the design is not randomised and is therefore theoretically open to confounding factors that may bias results: causation is not proven and the level or grade of evidence could be higher. A Cochrane review on the topic of early antibiotics in sepsis published in 2010 (Siddiqui and Razzak 2010) is not cited in any of the source documents but concludes that “we are unable to make a recommendation on the early or late use of broad spectrum antibiotics in adult patients with severe sepsis in the ED pre-ICU admission.” The authors concluded that there was a need for appropriately designed trials to address this question and that “…it makes sense…” to start antibiotics early but that this was based on “…anecdotal suboptimal evidence”. Again, the recommendation to give early antibiotics makes intuitive sense and is supported by some observational data but in the general context of a very limited evidence base on the topic. Much the same can be said for all the NELA recommendations; they are pragmatic, logical and based upon a hopefully balanced and unbiased judgement reflecting the best available evidence by practicing clinical experts.

Part of the problem restricting the current evidence base underpinning the NELA standards is the difficulty of conducting trails in emergency laparotomy patients specifically or in critically ill patients in general. Patients undergoing emergency laparotomy are a heterogeneous group with a range of surgical pathologies and exposed to a variety of clinical care processes: large numbers of patients may be required to separate signal from noise. The emergency context brings particular challenges to the consent process, but recent large trails in critical care show that these are not insurmountable. Randomisation of individual emergency laparotomy patients to different packages of care may be substantially undermined by washover of care practices within hospital wards and teams over time. The solution to this last problem may lie in less conventional research trial designs (see below).

Despite the complexities of doing clinical studies in patients undergoing emergency laparotomies, the evidence base is growing with one recent important publication and two other studies close to reporting results. None of these studies uses a classical parallel group randomisation of patients to intervention or control. A non-randomised “before and after” evaluation of implementation of best practice guidelines, the Emergency Laparotomy Pathway Quality Improvement Care (ELPQuiC) bundle, recruited 726 patients in four centres and published results in 2014 demonstrating a mortality reduction in the implementation phase (Huddart et al. 2015). The follow-on study for this pilot, a 2-year quality improvement project being conducted in 24 NHS hospital trusts in three Academic Health Sciences Networks (AHSNs) in southern England, will be completed in 2017 (Emergency Laparotomy Collaborative (ELC)). Finally, the hugely ambitious Enhanced Perioperative Care for High-risk patients (EPOCH) study (Pearse 2015), which has a planned sample size of 27,540 patients in 90 NHS Hospitals in a stepped wedge cluster randomised trial design, has recently completed recruitment and should report during 2016. The design of this study is important, overcoming the restrictions around protecting group allocation by gradually designating geographic clusters of hospitals to the invention over time, providing an effective method of evaluating the implementation of a package of care despite logistical constraints (Hemming et al. 2015).

## Conclusions

The current evidence base underpinning national standards around the care of patients undergoing emergency laparotomy is weak, in terms of both the quantity and grade of evidence. However, this situation is rapidly improving. The next 24 months will see important evidence emerging from EPOCH and the ELC. Clearer definition of what is best practice in this patient group will be of great value to clinicians and healthcare providers and may also pave the way for financial incentives for Trusts (Murray 2014), in a similar manner to those shown to improve care for patients having hip fracture surgery (Khan et al. 2013). The challenges of conducting clinical studies in this context should not prevent the development of a more solid evidence base to underpin standards and guide clinical care, through the conduct of appropriately designed and carefully conducted clinical trials.

## Abbreviations

NELA: National Emergency Laparotomy Audit
EL: Emergency laparotomy
ASAP: Hip Fracture Anaesthesia Sprint Audit of Practice
NCEPOD: National Confidential Enquiry into Patient Outcome and Death
ASGBI: Association of Surgeons of Great Britain and Ireland
RCS: Royal College of Surgeons of England
DoH: Department of Health
NHSE: National Health Service England
RCT: Randomised controlled trial
CQUIN: Commissioning for Quality and Innovation
ELPQuiC: Emergency Laparotomy Pathway Quality Improvement Care
AHSNs: Academic Health Sciences Networks
ELC: Emergency Laparotomy Collaborative
EPOCH: Enhanced Perioperative Care for High-risk patients
ELPQuiC: Emergency Laparotomy Pathway Quality Improvement Care
